# Supplementary material for: Adaptive divergence and post-zygotic barriers to gene flow between sympatric populations of a herbivorous mite
Source: Commun Biol. 2021 Jul 9;4:853. doi: 10.1038/s42003-021-02380-y (PMC8270941; doi:10.1038/s42003-021-02380-y)
Supplement: Supplementary file 2 — Supplementary information [file 42003_2021_2380_MOESM2_ESM.pdf]

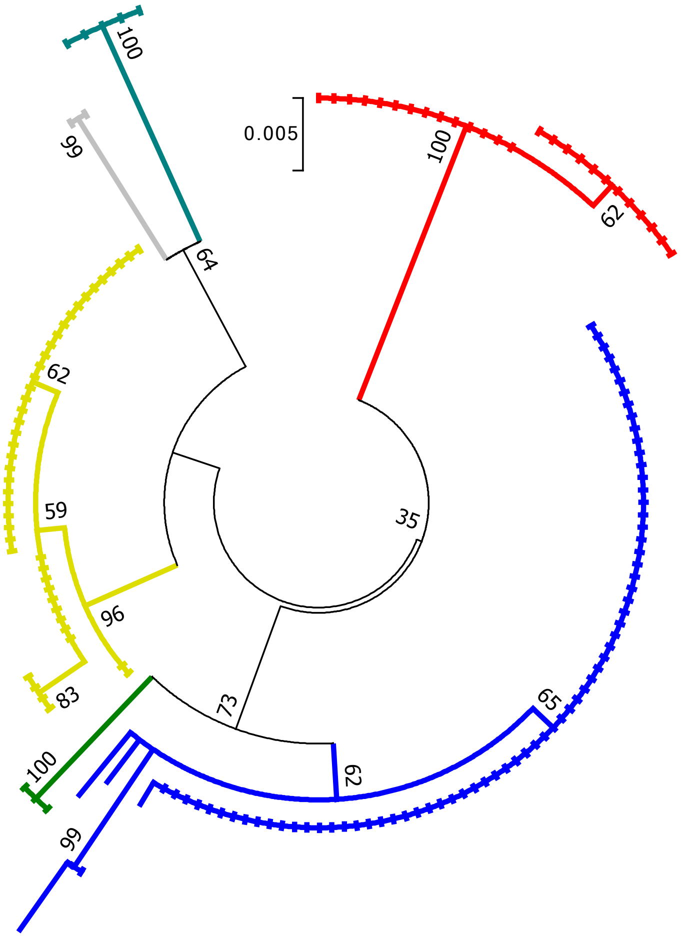

Supplementary Figure 1. Phylogenetic analysis of all the *CO1* haplotypes found in *Tetranychus urticae* individuals in the dune ecosystem of The Netherlands. The phylogenetic relation of 156 unique *CO1* haplotypes observed among all spider mite individuals from the Dutch coastal dunes collected over the 2015, 2016 and 2017 field seasons is shown. The tree is drawn to scale, with branch lengths measured as the number of substitutions per site. Clades with bootstrap values >60 were coloured and labelled as cytotypes 1 through 6. The most common and prevalent cytotypes were C1, C2 and C3 (in red, blue, and yellow, respectively), while C4, C5 and C6 (in green, turquoise, and grey, respectively) were rare and were not found every year in the field.

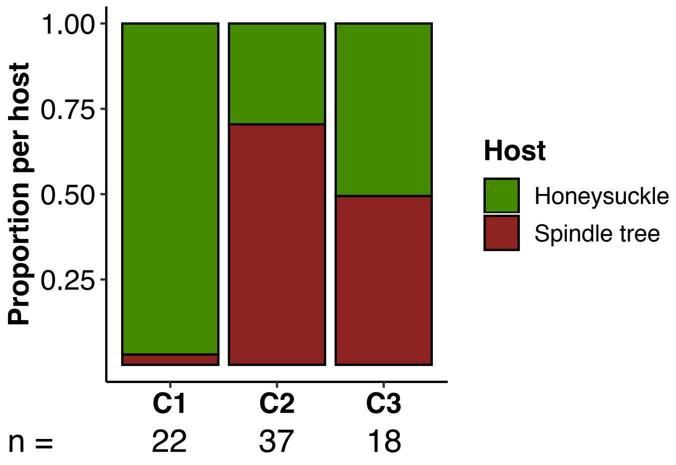

Supplementary Figure 2. Occurrence of three common *Tetranychus urticae* cytotypes on two common host species in the dune ecosystem of The Netherlands. The proportion of individuals (y-axis) belonging to each of three cytotypes, C1, C2 or C3, found on wild honeysuckle and spindle tree plants (legend, right) in the dune ecosystem of The Netherlands as summed over three consecutive field seasons (2015-17). The number of locations at which a cytotype occurred is shown underneath each cytotype (n).

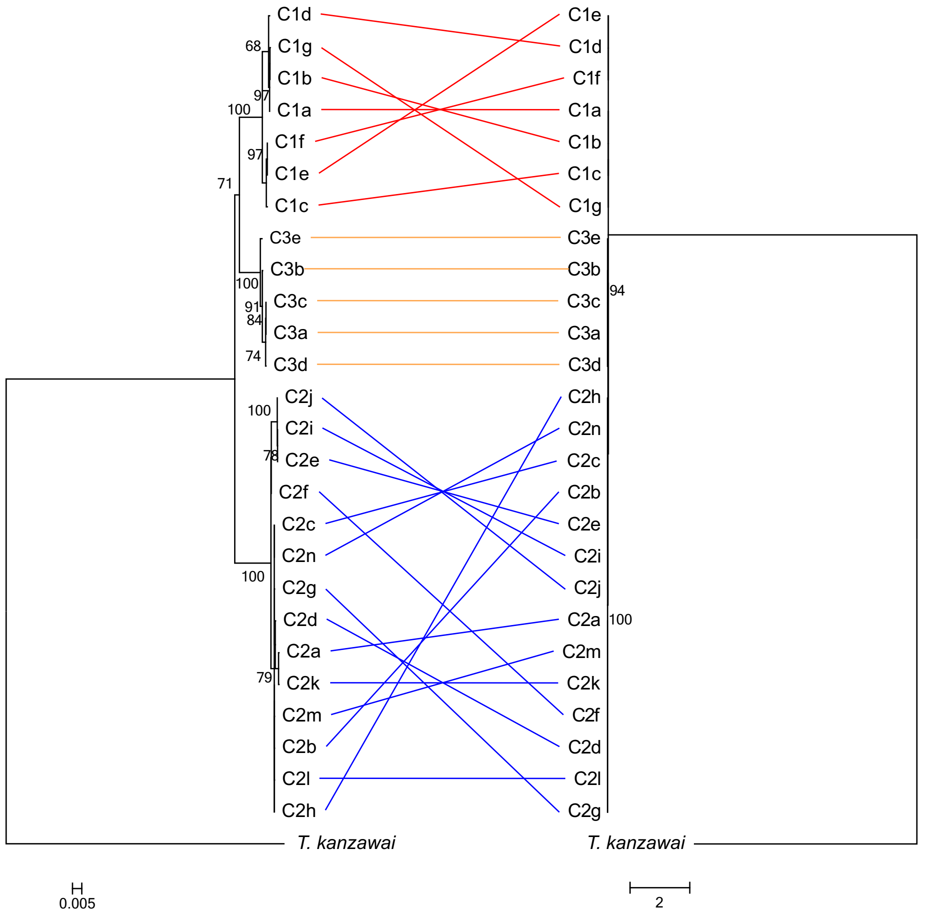

Supplementary Figure 3. Congruence between phylogenetic analyses based on *CO1* and full mitochondrial sequences of the field-derived *Tetranychus urticae* lines. Maximum likelihood analyses based on the *CO1* sequences (left) and on the optimal partitioning scheme of the 13 mitochondrial genes (right). The two scale bars represent the number of nucleotide substitutions per site. The lines linking the names of the *T. urticae* lines between the two phylogenies are coloured based on their cytotype (C1: red, C2: blue, C3: yellow). Only bootstraps ≥ 65 are shown.

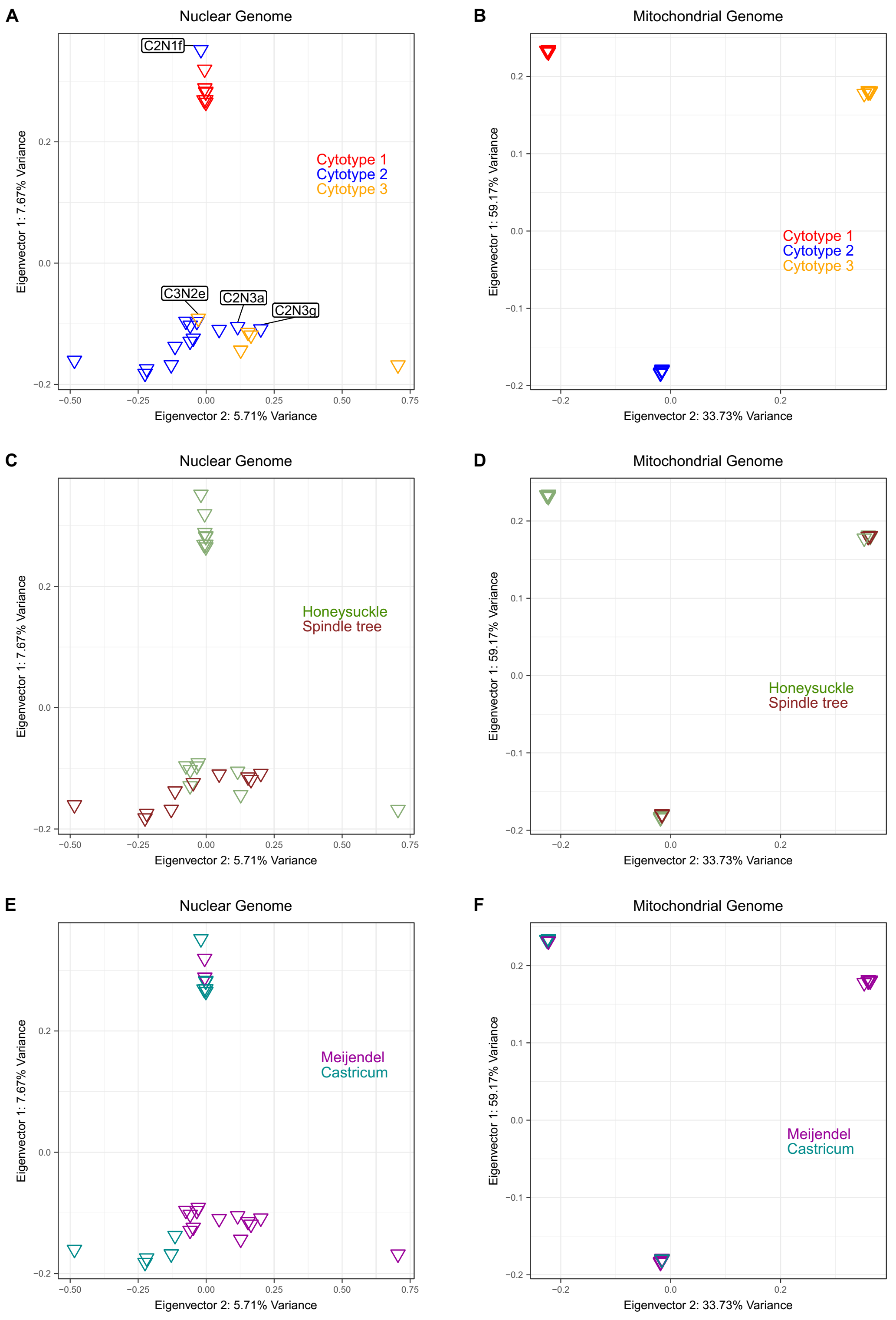

Supplementary Figure 4. PCA plots of the field-derived *Tetranychus urticae* lines. Principal component analyses for 26 field-derived lines based on nuclear and mitochondrial variation (left versus right) demarcated by cytotype, host plant species, and sampling location as indicated (A&B, C&D, and E&F, respectively). For panel A, each cyto-nuclear hybrid line is labelled individually in black.

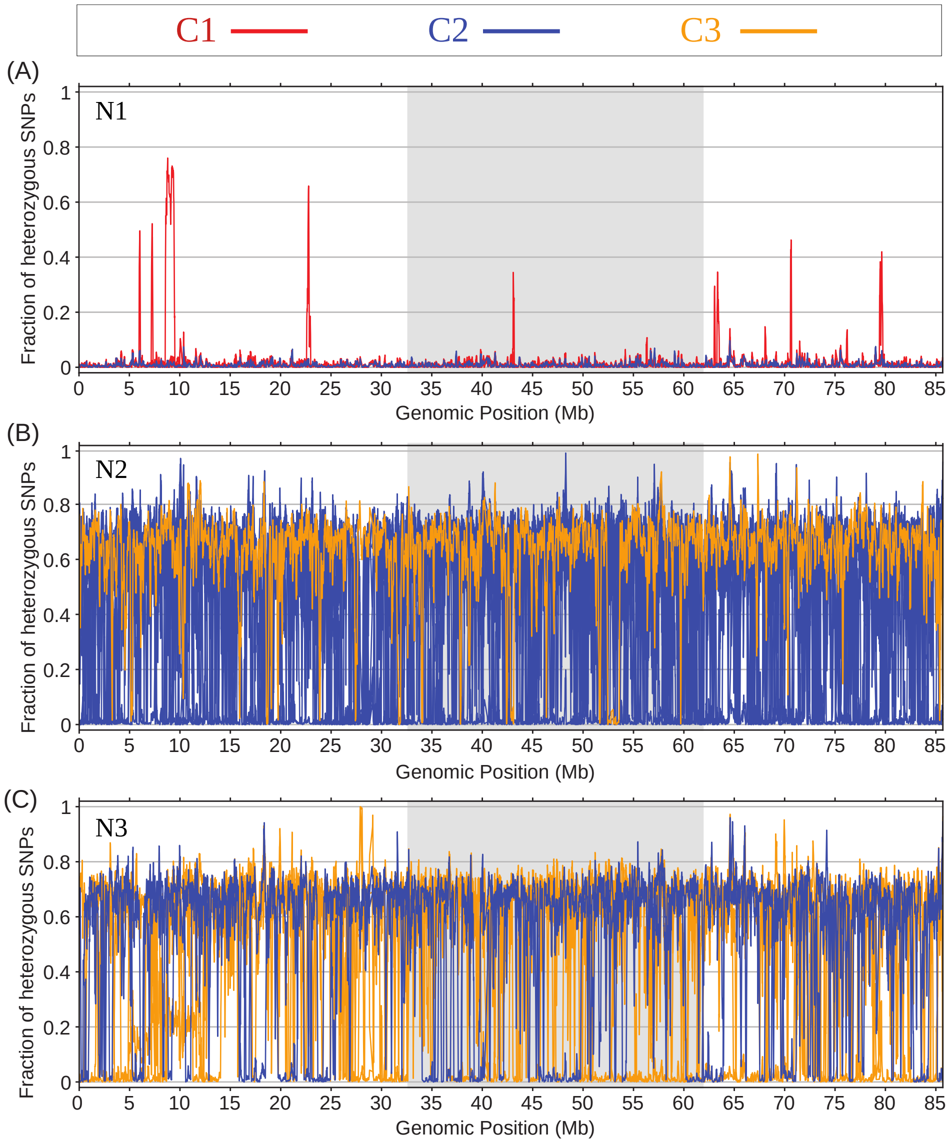

Supplementary Figure 5. Heterozygosity along the genome of field-derived *Tetranychus urticae* lines. The fraction of heterozygous (segregating) SNPs (y-axis) was plotted in 100kb sliding windows along the genome (cumulative genomic position on x-axis) for all lines and presented in a separate panel for each nucleotype: (A) N1, (B) N2, and (C) N3. The *T. urticae* lines were coloured based on their cytotype: C1 in red, C2 in blue, and C3 in orange.

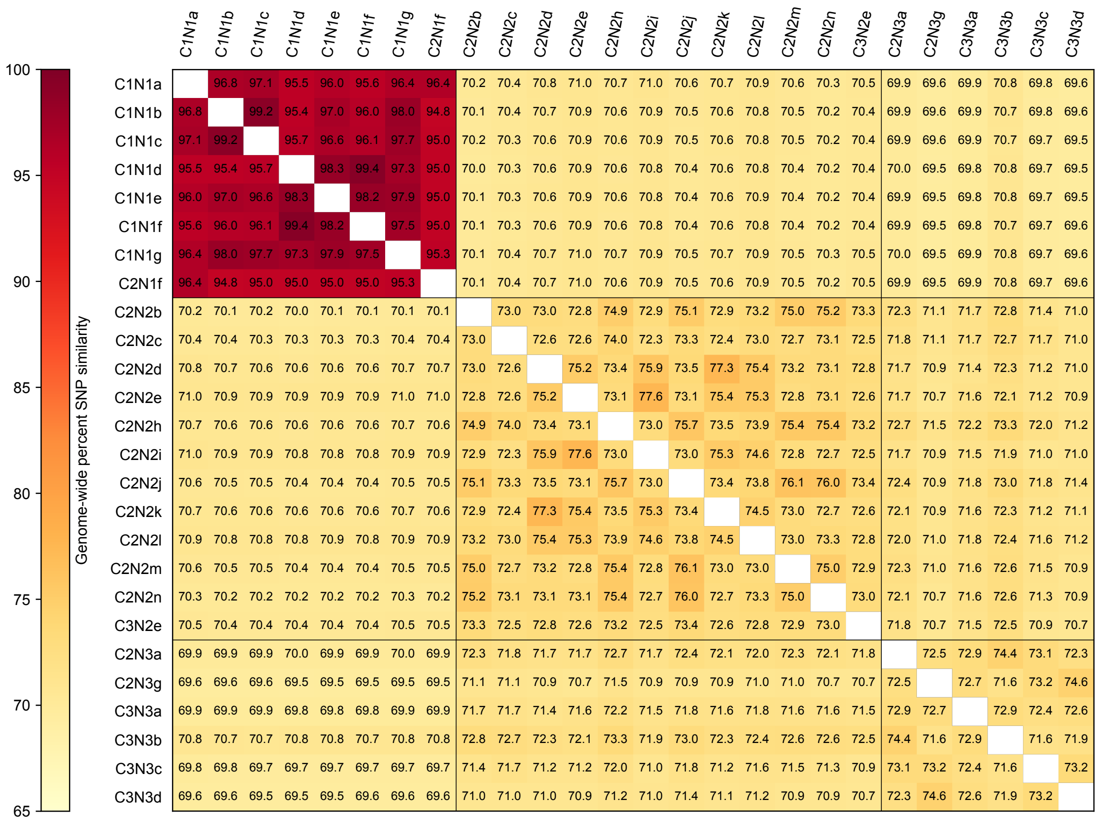

Supplementary Figure 6. Percentage of genome-wide SNP similarity between field-derived *Tetranychus urticae* lines. The lines are grouped by their nucleotype (N1, N2, or N3), from left to right and from top to bottom.

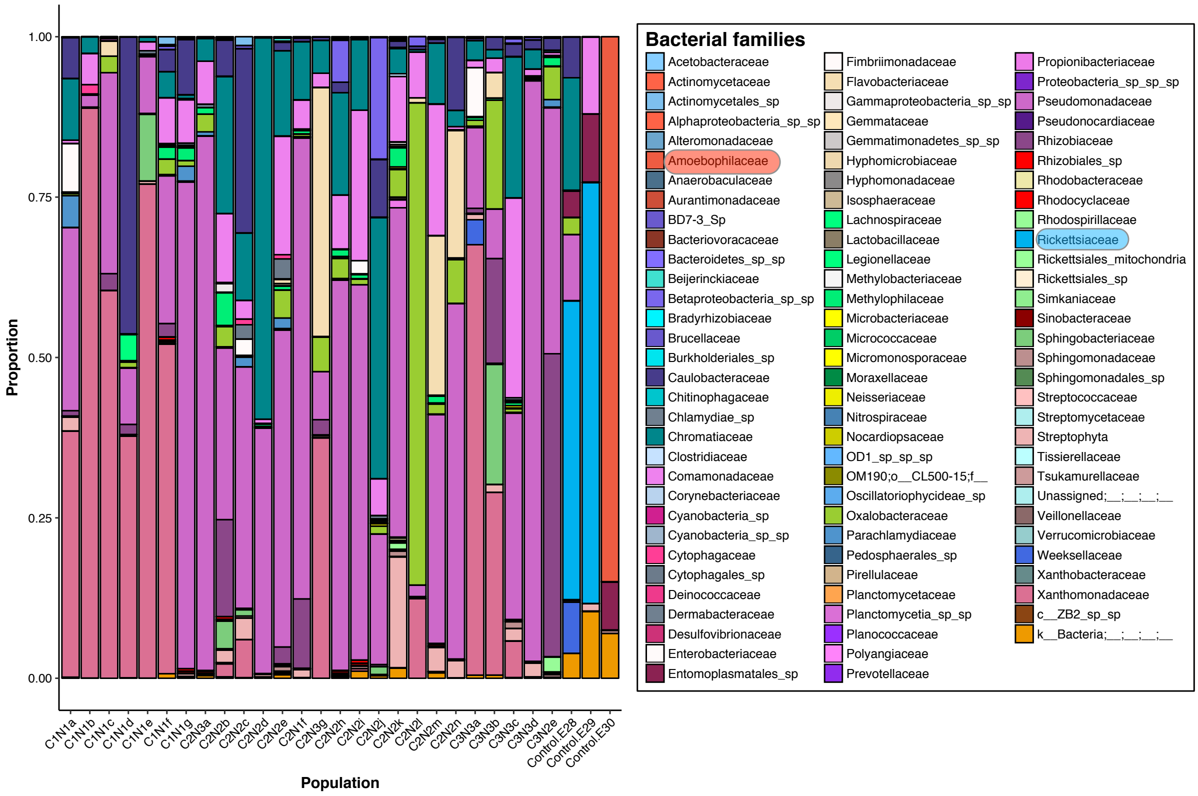

Supplementary Figure 7. Proportions of bacterial families associated with field-derived *Tetranychus urticae* lines. The proportion of 16S bacterial sequences (y-axis), identified to the family level, associated with the 26 iso-female lines of this study, and three laboratory lines (Controls E28, E29, E30) known to harbour common mite endosymbionts as positive controls (x-axis). In the legend, the families of *Wolbachia* and *Cardinium*, two known reproductive manipulating bacteria associated to arthropods, are highlighted in blue and red, respectively. These taxa were not present in the iso-female lines.

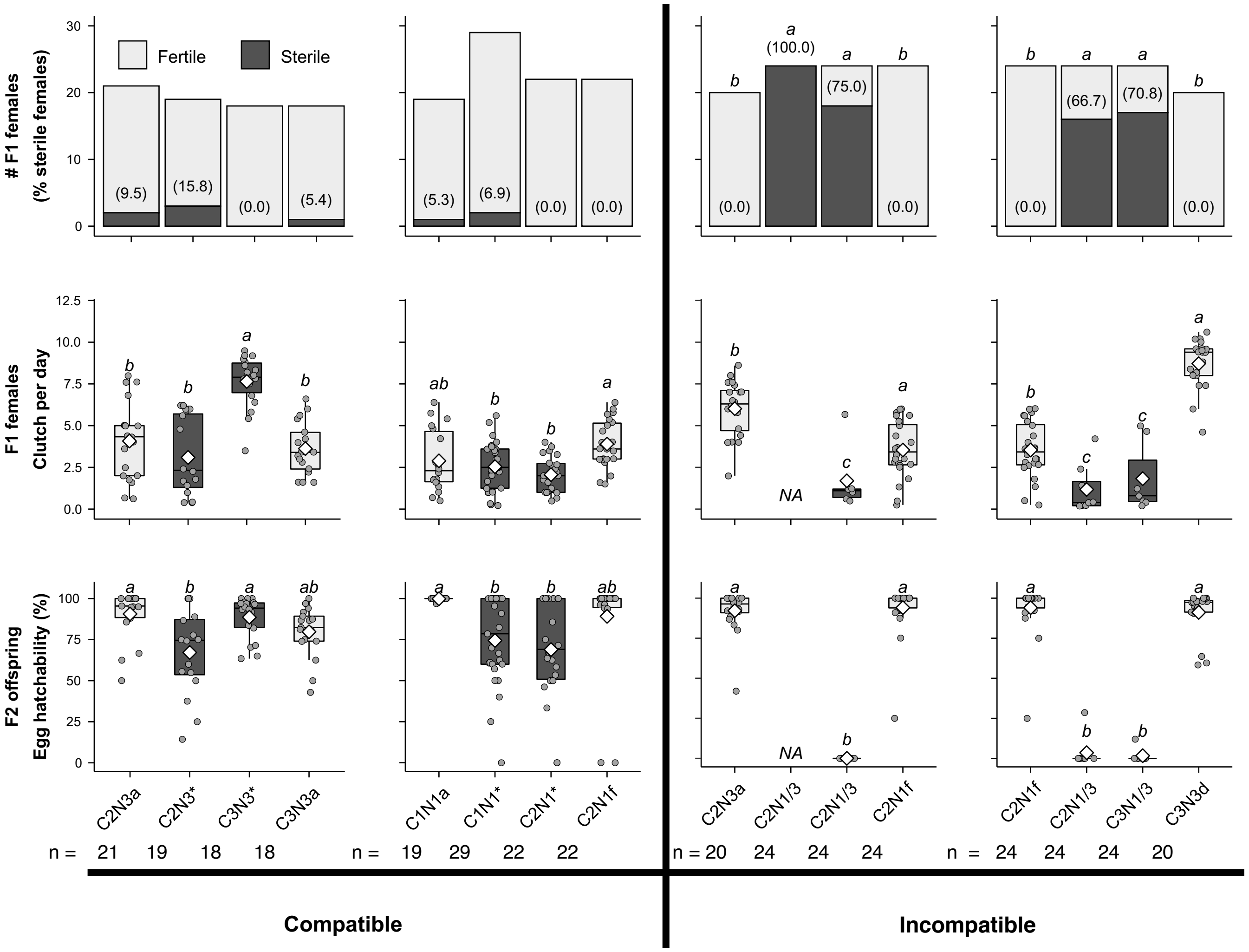

Supplementary Figure 8. Analyses of reproductive compatibility between selected field-derived *Tetranychus urticae* lines. Reciprocal crosses between lines hypothesised to be either compatible (same nucleotype) or incompatible (different nucleotypes) (♀X♂ in the x-axes of all panels). Fitness traits measured in individuals across two filial generations are presented on the y-axes of each row. F1 female clutch per day and F2 egg hatchability are presented in boxplots showing each data point (grey dots), the median (black line within the interquartile range box), the mean (white diamond) and the bottom 25% and the top 25% of the data values (whiskers); reciprocal ♀x♂ crosses are presented in black boxes and their respective controls in light grey boxes. F1 female sterility is presented as the number of females (fecund females in light grey and sterile females in dark grey) with the percentage of sterile females in parenthesis. Letters above bars and boxes represent significant differences within each panel. Chi-square and *F*-statistics, and p-values are specified in Supplementary Table 4.

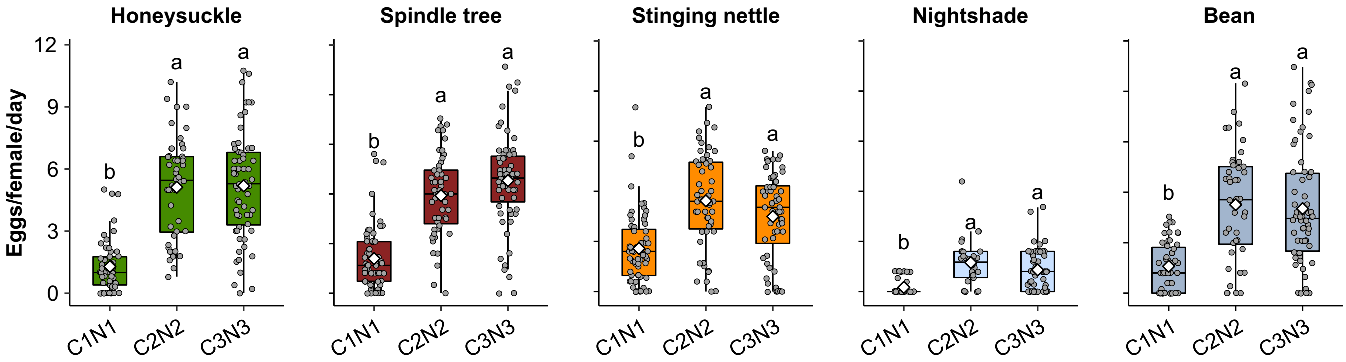

Supplementary Figure 9. Reproductive performance of field-derived *Tetranychus urticae* lines on multiple host species. The number of eggs per female per day (y-axes) are presented in boxplots showing each data point (grey dots), the median (black line within the interquartile range box), the mean (white diamond) and the bottom 25% and the top 25% of the data values (whiskers). Young mated females from 4 to 5 iso-female lines belonging to either C1N1, C2N2 or C3N3 were allowed to oviposit on leaf discs of either honeysuckle (*Lonicera peryclimenum*), spindle tree (*Euonymus europaeus*), stinging nettle (*Urtica sp.*), night-shade (*Solanum nigrum*) or common bean (*Phaseolus vulgaris* cv. Speedy). Significant differences, analysed with a linear mixed-effect model fitted to each host (α≤ 0.05), are represented by different letters above boxes according to a Tukey post-hoc test. *F*-statistics, and *p*-values are specified in Supplementary Table 6.

Supplementary Table 1. **Cytotype, nucleotype, label, native host, sampling site, field coordinates, SNP characteristics and heterozygosity of each field-derived *Tetranychus urticae* iso-female line**

Cytotype	Nucleotype	Line	Host plant	Site	Coordinates	Mean SNP coverage depth	% heterozygosity	No. SNPs
1	1	C1N1a	Honeysuckle	Meyendel	N52° 07.900' E4° 21.262'	79.99	0.95%	561576
1	1	C1N1b	Honeysuckle	Castricum	N52° 32.433' E4° 38.531'	82.38	0.90%	562328
1	1	C1N1c	Honeysuckle	Meyendel	N52° 07.815' E4° 19.860'	71.2	0.91%	562297
1	1	C1N1d	Honeysuckle	Castricum	N52° 32.617' E4° 37.612'	78.86	1.01%	562901
1	1	C1N1e	Honeysuckle	Castricum	N52° 32.867' E4° 38.288'	77.78	2.10%	565881
1	1	C1N1f	Honeysuckle	Castricum	N52° 32.984' E4° 38.524'	75.06	1.25%	563273
1	1	C1N1g	Honeysuckle	Castricum	N52° 32.542' E4° 38.623'	107.61	0.87%	561564
2	1	C2N1f	Honeysuckle	Castricum	N52° 32.365' E4° 38.472'	78.09	0.96%	563242
2	2	C2N2b	Spindle tree	Castricum	N52° 32.590' E4° 38.356'	86.32	47.45%	740588
2	2	C2N2c	Spindle tree	Meyendel	N52° 09.616' E4° 21.642'	78.05	53.18%	767328
2	2	C2N2d	Honeysuckle	Meyendel	N52° 07.871' E4° 20.385'	84.32	47.47%	727628
2	2	C2N2e	Honeysuckle	Meyendel	N52° 07.871' E4° 20.451'	82.09	51.21%	753045
2	2	C2N2h	Spindle tree	Castricum	N52° 33.049' E4° 38.705'	89.32	46.62%	729987
2	2	C2N2i	Honeysuckle	Meyendel	N52° 07.871' E4° 20.451'	94.66	54.16%	773754
2	2	C2N2j	Spindle tree	Castricum	N52° 32.643' E4° 37.787'	80.53	51.72%	756568
2	2	C2N2k	Honeysuckle	Meyendel	N52° 07.871' E4° 20.385'	77.25	55.00%	771338
2	2	C2N2l	Spindle tree	Meyendel	N52° 07.815' E4° 19.768'	70.31	55.35%	771722
2	2	C2N2m	Spindle tree	Castricum	N52° 32.587' E4° 37.806'	94.94	32.94%	668879
2	2	C2N2n	Spindle tree	Castricum	N52° 32.593' E4° 38.759'	79.44	30.33%	662172
3	2	C3N2e	Honeysuckle	Meyendel	N52° 07.905' E4° 21.384'	76.31	63.53%	816463
2	3	C2N3a	Honeysuckle	Meyendel	N52° 09.468' E4° 21.691'	80.78	53.08%	780419
2	3	C2N3g	Spindle tree	Meyendel	N52° 09.536' E4° 22.571'	82.77	54.83%	807683
3	3	C3N3a	Honeysuckle	Meyendel	N52° 09.546' E4° 21.655'	73.5	33.78%	688540
3	3	C3N3b	Honeysuckle	Meyendel	N52° 09.428' E4° 21.628'	81.04	45.91%	737728
3	3	C3N3c	Spindle tree	Meyendel	N52° 09.625' E4° 22.207'	66.33	59.61%	828189
3	3	C3N3d	Spindle tree	Meyendel	N52° 09.536' E4° 22.571'	79.29	58.79%	831822

Supplementary Table 2. Fitness traits of parents (P0) and resulting hybrid F1 offspring between field-derived iso-female lines of *Tetranychus urticae* belonging to sympatric genotype groups C1N1, C2N2, C3N3[1]

Cross	N	P0 Female sterility[2]	P0 females Days alive[3]	N	Clutch size (per day)[3]	F1 Egg viability[2]	N	F1 Juvenile survival[2]	Sons (per day) [3]	Daughters (per day) [3]	N	F1 sex ratio[2,*]	N	F1 adults per clutch[2]
**C1N1**	43	9.30 ± 4.23	2.98 ± 0.18 b	39	2.13 ± 0.22 b	94.17 ± 1.68	39	84.81 ± 3.06	0.53 ± 0.06 b	1.23 ± 0.16 b	39	60.52 ± 5.01	39	84.81 ± 3.06
**C2N2**	29	0.00 ± 0.64	3.28 ± 0.28 b	29	5.25 ± 0.61 a	97.03 ± 0.97	29	83.46 ± 3.66	1.08 ± 0.17 a	3.49 ± 0.46 a	29	71.40 ± 4.67	29	83.46 ± 3.66
**C3N3**	53	3.77 ± 2.58	4.47 ± 0.12 a	51	6.82 ± 0.41 a	92.42 ± 1.88	51	80. 08 ± 2.51	1.56 ± 0.16 a	3.96 ± 0.35 a	51	68.31 ± 3.02	51	80.08 ± 2.51
Stats.		$\chi^2_2 = 3.49$<br>$p = 0.17$	$F_{2, 43.98} = 21.68$<br>$\mathbf{p < 0.001}$		$F_{2, 49.05} = 29.99$<br>$\mathbf{p < 0.001}$	$F_{2, 56.46} = 0.78$<br>$p = 0.46$		$F_{2, 53.62} = 0.42$<br>$p = 0.66$	$F_{2, 40.83} = 11.90$<br>$\mathbf{p < 0.001}$	$F_{2, 56.39} = 15.48$<br>$\mathbf{p < 0.001}$		$F_{2, 53.10} = 0.77$<br>$p = 0.47$		$F_{2, 53.62} = 0.42$<br>$p = 0.66$
**C1N1**	43	9.30 ± 4.23 a	2.98 ± 0.18	39	2.13 ± 0.23 b	94.17 ± 1.68	39	84.81 ± 3.06	0.53 ± 0.06 b	1.23 ± 0.16 b	39	60.52 ± 5.01	39	84.81 ± 3.06
**1x2**	38	0.00 ± 0.43 b	3.45 ± 0.18	38	2.58 ± 0.30 b	93.43 ± 2.80	37	84.78 ± 3.28	0.51 ± 0.06 b	1.79 ± 0.24 b	36	71.04 ± 3.48	38	82.54 ± 3.90
**2x1**	34	0.00 ± 0.50 b	3.62 ± 0.22	34	5.49 ± 0.58 a	93.13 ± 3.23	33	83.73 ± 2.64	1.78 ± 0.27 a	2.90 ± 0.29 a	33	66.41 ± 2.82	34	81.26 ± 3.55
**C2N2**	29	0.00 ± 0.64 b	3.28 ± 0.22	29	5.25 ± 0.61 a	97.03 ± 0.97	29	83.46 ± 3.66	1.09 ± 0.17 ab	3.49 ± 0.46 a	29	71.40 ± 4.67	29	83.46 ± 3.66
Stats.		$\chi^2_3 = 9.66$<br>$\mathbf{p = 0.02}$	$F_{3, 128.06} = 1.73$<br>$p = 0.16$		$F_{3, 70.49} = 13.58$<br>$\mathbf{p < 0.001}$	$F_{3, 129.32} = 0.50$<br>$p = 0.68$		$F_{3, 59.82} = 0.09$<br>$p = 0.97$	$F_{3, 74.83} = 8.67$<br>$\mathbf{p < 0.001}$	$F_{3, 59.03} = 11.67$<br>$\mathbf{p < 0.001}$		$F_{3, 56.65} = 1.29$<br>$p = 0.29$		$F_{3, 52.07} = 0.24$<br>$p = 0.87$
**C1N1**	43	9.30 ± 4.23	2.98 ± 0.18 b	39	2.13 ± 0.23 b	94.17 ± 1.68 a	39	84.81 ± 3.06	0.53 ± 0.06 c	1.23 ± 0.16 c	39	60.52 ± 5.01 a	39	84.81 ± 3.06
**1x3**	51	12.00 ± 4.32	2.96 ± 0.18 b	44	2.89 ± 0.29 b	81.86 ± 4.47 b	42	77.36 ± 3.94	0.59 ± 0.07 c	1.73 ± 0.25 c	41	57.73 ± 5.70 ab	44	73.84 ± 4.49
**3x1**	54	5.56 ± 3.04	4.26 ± 0.15 a	51	6.97 ± 0.41 a	91.46 ± 1.84 a	51	78.04 ± 2.50	3.33 ± 0.25 a	2.26 ± 0.20 b	51	37.42 ± 2.60 b	51	78.69 ± 2.53
**C3N3**	53	3.77 ± 2.58	4.47 ± 0.12 a	51	6.82 ± 0.41 a	92.42 ± 1.88 a	51	80. 08 ± 2.51	1.56 ± 0.16 b	3.96 ± 0.35 a	51	68.31 ± 3.02 a	51	80.08 ± 2.51
Stats.		$\chi^2_3 = 3.02$<br>$p = 0.39$	$F_{3, 195.3} = 23.25$<br>$\mathbf{p < 0.001}$		$F_{3, 60.54} = 49.69$<br>$\mathbf{p < 0.001}$	$F_{3, 66.94} = 4.24$<br>$\mathbf{p = 0.008}$		$F_{3, 70.69} = 0.87$<br>$p = 0.46$	$F_{3, 177.9} = 67.67$<br>$\mathbf{p < 0.001}$	$F_{3, 77.84} = 15.92$<br>$\mathbf{p < 0.001}$		$F_{3, 82.50} = 8.16$<br>$\mathbf{p < 0.001}$		$F_{3, 73.68} = 1.49$<br>$p = 0.22$
**C2N2**	29	0.00 ± 0.64	3.28 ± 0.28 b	29	5.25 ± 0.61 b	97.03 ± 0.97	29	83.46 ± 3.66	1.09 ± 0.17 b	3.49 ± 0.46	29	71.40 ± 4.67 a	29	83.46 ± 3.66
**2x3**	42	0.00 ± 0.37	3.93 ± 0.19 ab	42	5.30 ± 0.45 b	94.40 ± 1.14	42	88.21 ± 1.71	1.06 ± 0.09 b	3.64 ± 0.37	42	72.19 ± 3.39 a	42	88.21 ± 1.71
**3x2**	32	0.00 ± 0.55	4.31 ± 0.19 a	32	7.99 ± 0.38 a	97.13 ± 1.19	32	87.27 ± 1.88	3.26 ± 0.33 a	3.82 ± 0.35	32	54.42 ± 4.10 b	32	87.27 ± 1.88
**C3N3**	53	3.77 ± 2.58	4.47 ± 0.12 a	51	6.82 ± 0.41 ab	92.42 ± 1.88	51	80.08 ± 2.51	1.56 ± 0.16 b	3.96 ± 0.35	51	68.31 ± 3.02 a	51	80.08 ± 2.51
Stats.		$\chi^2_3 = 3.94$<br>$p = 0.27$	$F_{3, 152} = 7.90$<br>$\mathbf{p < 0.001}$		$F_{3, 52.76} = 7.28$<br>$\mathbf{p < 0.001}$	$F_{3, 50.38} = 0.43$<br>$p = 0.73$		$F_{3, 62.42} = 0.49$<br>$p = 0.69$	$F_{3, 32.94} = 20.39$<br>$\mathbf{p < 0.001}$	$F_{3, 53.19} = 1.59$<br>$p = 0.20$		$F_{3, 45.36} = 4.80$<br>$\mathbf{p = 0.005}$		$F_{3, 62.42} = 0.49$<br>$p = 0.69$

1: Variables that differed significantly ($p \leq 0.05$) from their respective intra-line controls in the linear-mixed models fitted are marked with different appended letters within each panel (column by rows between thin black lines), according to a Tukey post-hoc test (see *Methods*). 2: Average percentage ± SEM. 3: Average mean ± SEM. *: tertiary sex ratio = females/adult offspring. N columns: sample size for columns on their right-side. Chi-square (χ^2) or *F*-statistic values, and *p* values are presented underneath each panel, with the number of degree of freedoms in the subscript. Denominator degrees of freedom for *F*-statistics of linear mixed models obtained with a Satterthwaite approximation.

Supplementary Table 3. Fitness traits of the F1 virgin females resulting from parental crosses between field-derived iso-female lines of *Tetranychus urticae* belonging to genotype groups C1N1, C2N2, C3N3, and fitness traits of their F2 male offspring[1]

Cross	N	F1 Female sterility[2]	F1 females Days alive[3]	N	Clutch size (per day) [3]	F2 egg viability [3]	N	F2 juvenile survival [3]	Sons (per day) [2]	N	F2 adults per clutch [3]
**C1N1**	51	19.61 ± 4.99	3.60 ± 0.22	41	3.42 ± 0.27 b	96.93 ± 1.18	41	96.41 ± 1.34 a	3.21 ± 0.26 b	41	93.38 ± 1.67 a
**C2N2**	38	2.63 ± 2.60	3.90 ± 0.24	37	5.03 ± 0.36 ab	88.34 ± 3.20	37	95.01 ± 1.38 a	4.25 ± 0.36 a	37	84.12 ± 3.31 b
**C3N3**	52	15.38 ± 4.61	4.35 ± 0.18	44	4.96 ± 0.39 a	87.04 ± 2.49	44	89.59 ± 1.91 b	4.05 ± 0.39 ab	44	78.67 ± 3.08 b
Stats.		$\chi^2_2 = 5.64$<br>$p = 0.06$	$F_{2,\ 43.96} = 2.28$<br>$p = 0.11$		$F_{2,\ 37.56} = 7.17$<br>**$p = 0.002$**	$F_{2,\ 37.56} = 7.17$<br>**$p = 0.002$**		$F_{2,\ 43.15} = 3.58$<br>**$p = 0.04$**	$F_{2,\ 41.28} = 3.34$<br>**$p = 0.05$**		$F_{2,\ 38.93} = 6.37$<br>**$p = 0.004$**
**C1N1**	51	19.61 ± 4.99 ab	3.60 ± 0.22 c	41	3.42 ± 0.27 b	96.93 ± 1.18 a	41	96.41 ± 1.34 a	3.21 ± 0.26 a	41	93.38 ± 1.67 a
**1x2**	41	26.83 ± 5.93 a	4.61 ± 0.11 a	30	2.46 ± 0.31 b	2.63 ± 1.15 c	7	78.57 ± 14.87 ab	0.27 ± 0.06 b	30	1.96 ± 0.83 b
**2x1**	35	11.43 ± 5.08 ab	4.43 ± 0.21 ab	31	3.27 ± 0.40 b	8.19 ± 2.14 b	15	74.44 ± 8.47 b	0.42 ± 0.11 b	31	5.29 ± 3.31 b
**C2N2**	38	2.63 ± 2.60 b	3.90 ± 0.24 bc	37	5.03 ± 0.36 a	88.34 ± 3.20 a	37	95.01 ± 1.38 a	4.25 ± 0.36 a	37	84.12 ± 3.31 a
Stats.		$\chi^2_3 = 9.78$<br>**$p = 0.02$**	$F_{3,\ 73.01} = 8.53$<br>**$p < 0.001$**		$F_{3,\ 63.82} = 11.2$<br>**$p < 0.001$**	$F_{3,\ 126.4} = 392.8$<br>**$p < 0.001$**		$F_{3,\ 12.82} = 5.99$<br>**$p = 0.009$**	$F_{3,\ 95.77} = 41.95$<br>**$p < 0.001$**		$F_{3,\ 127.5} = 486.9$<br>**$p < 0.001$**
**C1N1**	51	19.61 ± 4.99 ab	3.60 ± 0.22 b	41	3.42 ± 0.27 ab	96.93 ± 1.18 a	41	96.41 ± 1.34 a	3.21 ± 0.26 a	41	93.38 ± 1.67 a
**1x3**	48	39.58 ± 5.49 a	4.84 ± 0.12 a	29	2.34 ± 0.30 bc	2.85 ± 1.30 b	5	70.00 ± 20.00 b	0.44 ± 0.17 b	29	2.32 ± 1.16 c
**3x1**	57	38.60 ± 5.06 a	4.38 ± 0.16 a	35	1.96 ± 0.26 c	1.10 ± 0.67 b	3	66.67 ± 33.33 b	0.18 ± 0.10 b	35	0.86 ± 0.63 c
**C3N3**	52	15.38 ± 4.61 b	4.35 ± 0.18 a	44	4.96 ± 0.39 a	87.04 ± 2.49 a	44	89.59 ± 1.91 a	4.05 ± 0.39 a	44	78.67 ± 3.08 b
Stats.		$\chi^2_3 = 12.15$<br>**$p = 0.007$**	$F_{3,\ 83.54} = 8.81$<br>**$p < 0.001$**		$F_{3,\ 57.96} = 21.8$<br>**$p < 0.001$**	$F_{3,\ 145} = 964.04$<br>**$p < 0.001$**		$F_{3,\ 89} = 8.34$<br>**$p < 0.001$**	$F_{3,\ 55.31} = 11.19$<br>**$p < 0.001$**		$F_{3,\ 78.94} = 444$<br>**$p < 0.001$**
**C2N2**	38	2.63 ± 2.60	3.90 ± 0.24	37	5.03 ± 0.36	88.34 ± 3.20 a	37	95.01 ± 1.38 a	4.25 ± 0.36 a	37	84.12 ± 3.31 a
**2x3**	36	5.56 ± 3.74	4.63 ± 0.21	34	6.41 ± 0.50	49.04 ± 4.03 b	33	85.72 ± 3.09 b	2.95 ± 0.38 ab	34	42.78 ± 3.88 b
**3x2**	36	13.89 ± 5.37	4.41 ± 0.23	31	6.95 ± 0.48	23.10 ± 4.80 c	26	91.43 ± 2.26 ab	2.05 ± 0.46 b	31	20.67 ± 4.38 c
**C3N3**	52	15.38 ± 4.61	4.35 ± 0.18	44	4.96 ± 0.39	87.04 ± 2.49 a	44	89.59 ± 1.90 ab	4.05 ± 0.39 a	44	78.67 ± 3.08 a
Stats.		$\chi^2_3 = 5.42$<br>$p = 0.14$	$F_{3,\ 65.45} = 2.36$<br>$p = 0.08$		$F_{3,\ 142} = 2.50$<br>$p = 0.06$	$F_{3,\ 126.5} = 71.45$<br>**$p < 0.001$**		$F_{3,\ 66.63} = 3.03$<br>**$p = 0.04$**	$F_{3,\ 133.57} = 6.43$<br>**$p < 0.001$**		$F_{3,\ 133.7} = 78.16$<br>**$p < 0.001$**

1: Variables that differed significantly ($p \leq 0.05$) from their respective intra-line controls in the linear-mixed models fitted are marked with different appended letters within each panel (columns by rows between thin black lines), according to a Tukey post-hoc test (see *Methods*). 2: Average percentage ± SEM. 3: Average mean ± SEM. N columns: sample size for columns on their right-side. Chi-square (χ^2) or *F*-statistic values, and *p* values are presented underneath each panel, with the number of degree of freedoms in the subscript. Denominator degrees of freedom for *F*-statistics of linear mixed models obtained with a Satterthwaite approximation.

Supplementary Table 4. Chi-square, *F*-statistics, degrees of freedom and *p*-values of the linear mixed models applied to the fitness traits of hybrids between compatible and incompatible mite lines

	Female sterility	Clutch size per day	F2 egg hatchability
**Compatible crosses**			
**C2N3a VS. C3N3a**	$\chi^2_3 = 3.38$<br>$p = 0.34$	$F_{3, 26.43} = 16.09$<br>**$p < 0.001$**	$F_{3, 66} = 6.07$<br>**$p = 0.001$**
**C1N1a VS. C2N1f**	$\chi^2_3 = 2.94$<br>$p = 0.40$	$F_{3, 85} = 6.23$<br>**$p < 0.001$**	$F_{3, 20.99} = 5.76$<br>**$p = 0.005$**
**Incompatible crosses**			
**C2N3a VS. C2N1f**	$\chi^2_3 = 73.86$<br>**$p < 0.001$**	$F_{3, 47} = 18.87$<br>**$p < 0.001$**	$F_{3, 47} = 311.61$<br>**$p < 0.001$**
**C2N1f VS. C3N3d**	$\chi^2_3 = 47.26$<br>**$p < 0.001$**	$F_{3, 55} = 44.87$<br>**$p < 0.001$**	$F_{3, 15.23} = 217.11$<br>**$p < 0.001$**

Supplementary Table 5. *F*-statistics, degrees of freedom and *p*-values of the linear mixed models applied to the juvenile survival of spider mite lines on honeysuckle

Day	C1N1a vs. C2N3a	C1N1d vs. C2N2d	C1N1e vs. C2N2b
**Day 5**	$F_{1, 10} = 3.97$<br>p = 0.07	$F_{1, 10} = 0.80$<br>p = 0.39	$F_{1, 10} = 3.64$<br>p = 0.09
**Day 7**	$F_{1, 10} = 11.18$<br>**p = 0.007**	$F_{1, 10} = 23.39$<br>**p < 0.001**	$F_{1, 10} = 16.67$<br>**p = 0.002**
**Day 9**	$F_{1, 10} = 16.44$<br>**p = 0.002**	$F_{1, 10} = 10.42$<br>**p = 0.009**	$F_{1, 10} = 11.99$<br>**p =0.006**
**Day 11**	$F_{1, 10} = 22.13$<br>**p < 0.001**	$F_{1, 10} = 5.12$<br>**p = 0.05**	$F_{1, 10} = 9.42$<br>**p = 0.01**
**Day 13**	$F_{1, 10} = 18.32$<br>**p = 0.002**	$F_{1, 10} = 0.29$<br>p = 0.60	$F_{1, 10} = 6.35$<br>**p = 0.03**

Supplementary Table 6. *F*-statistics, degrees of freedom and *p*-values of the linear mixed models applied to the reproductive performance of spider mite genotypes on multiple host plant species.

Host plant	F-value	p-value
**Honeysuckle**	$F_{2, 11.28} = 24.59$	**< 0.001**
**Spindle tree**	$F_{2, 10.93} = 14.16$	**< 0.001**
**Stinging nettle**	$F_{2, 10.55} = 7.00$	**0.01**
**Nightshade**	$F_{2, 11.71} = 9.09$	**0.004**
**Bean**	$F_{2, 10.98} = 6.00$	**0.02**

Supplementary Note

Supplementary Note 1: *Individual DNA extraction and CO1 amplification*

Individual *Tetranychus urticae* mites were placed in PCR strip tubes and crushed directly with a pipette tip in 20µl of TE buffer (10mM Trish-HCL, 100mM NaCl, 1mM EDTA [ph8]) and 1µl of Proteinase K (20mg/µl); samples were incubated for 30mins at 37°C, followed by inactivation of Proteinase K for 7mins at 95°C; DNA was then stored at -20°C until downstream analyses. Each sample was genotyped by analysing single nucleotide polymorphisms (SNPs) within the Folmer fragment (Folmer *et al.* 1994), which is a ~700 base pair (bp) stretch within the mitochondrially encoded gene *cytochrome oxidase subunit 1* (CO1). The forward primer (LCO1490) was 5'-GGTCAACAAATCATAAAGATATTGG-3'; the reverse primer (HCO2198) was 5'-TAAACTTCAGGGTGACCAAAAAATCA-3'. The PCR reaction was performed using two different protocols. In 2015 and 2016, 1µl of DNA extract was added to a 19µl mix comprised of 9.4µl purified water, 4.0 µl of 5X Hot start polymerase buffer, 4.0 µl of 1mM dNTPs, 0.6 µl of each primer [10 µM] and 0.4 µl of Hot start polymerase. For this mix, PCR conditions were: 30secs at 98°C, and 34 cycles of 10secs at 98°C, 10secs at 48°C, 15secs at 72°C and a final step at 72°C for 60secs. In 2017, the PCR reaction instead contained 3µl mite DNA in a 25µl mix of 11.0µl water, 3.75 µl of 10x *Taq* buffer, 5.0 µl of 1mM dNTP's, 1.25 of bovine serum albumin 10mM 0.4µl of each primer [10µM] and 0.2µl *Taq* polymerase 5U. Four µl of each PCR product was checked in a 1% agarose gel stained with Midori green.

Supplementary Note 2: Pooled DNA extraction

Between 400 and 800 adult females from the first laboratory generation of each iso-female line (26 in total) were aspirated from bean leaves using a vacuum and were collected in a single Eppendorf tube. The tubes were flash frozen in liquid nitrogen and stored at -80°C before DNA extraction. Purified DNA was obtained by homogenising each sample in a mix of 100µL SDS buffer (200mM Tris-HCl, 400mM NaCl, 10mM EDTA, 2% SDS at pH 8.2), 15µl Proteinase K and 3µl of RNase A. After incubation at 60°C for 2 hours in a water bath, 3µl RNase A were added to each sample, and incubated at 37°C for 1h45mins in a Thermomixer at 300rpm. The homogenate was moved to a new 2ml tube and an equal volume (~800µl) of a Phenol: Chloroform: isoamyl alcohol solution (25:24:1) was added and shaken manually for 1min. After centrifugation for 5m at ~14 000rpm, the aqueous layer containing the DNA was transferred to a new safe-lock Eppendorf 1.5ml tube, at which point ~450µl of ice-cold isopropanol was added, and the resulting solution was mixed by slowly inverting the tube. After incubation for 15mins at room temperature, the tubes were centrifuged at maximum speed for 45mins at 4°C. The supernatant was removed with a pipette and the resulting pellet was washed two consecutive times with 500µl of ice-cold ethanol (70%). After washing, the ethanol was further removed by centrifuging and collecting all droplets, and by allowing the DNA pellet to dry for 3mins. The pellet was eluted in 32µl of standard elution buffer and resolved overnight at 4°C. The quality, purity, and amount of DNA per sample were first assessed by Nanodrop measurements and by visual assessment in a 1% agarose gel (ran for

~45mins at 50V). An aliquot of 3µl per sample was diluted 10 times in double-distilled water and used for downstream PCR analyses; the undiluted DNA was used for whole-genome sequencing (WGS).

Supplementary Note 3: Endosymbiont presence diagnosis

Aliquots of purified DNA (1:10; see above) were used to diagnose the presence of three common endosymbionts in each of the 26 iso-female lines created from field samples within one generation after being established in the laboratory. The presence of a bacterial symbiont was assessed in two ways. First, we amplified marker genes previously reported for known arthropod endosymbionts: (1) the *Wolbachia wsp* gene using primers 81F and 691R (Zhou 1998), (2) the *Cardinium CLO* gene using primers F1 and R1 [1], (3) the *Spiroplasma dnaA* gene using primers ApDNaAF1 and ApDNAaR1 [2]. For *wsp* and *dnaA* amplification, we used a mix of 12.25µl of ddH20, 2.5µl of 10x buffer, 5µl of 1mM dNTPs, 1.25µl of BSA 10mM, 0.4µl of 10uM for each primer, 0.2µl of DreamTaq polymerase (ThermoFisher) (5U) and 3µL of DNA template, for a total volume of 25µl per sample. The protocol used for these two genes started with 2mins denaturation at 95°C, followed by 35 cycles of 30secs at 95°C, 30secs of annealing at 51°C (52°C for dnaA), 60secs at 72°C, and a final step for 4mins at 72°C. For *CLO* amplification, we used a mix of 13.5µl of ddH20, 2.5µl of 10x buffer, 5µl of 1mM dNTP's, 0.4µl of 10µM for each primer, 0.2µl of DreamTaq polymerase (ThermoFisher, 5U) and 3µl of DNA template, for a total volume of 25µl per sample. The protocol for *CLO* amplification started with 2mins denaturation at 94°C, followed by 35 cycles of 40secs at 94°C, 40secs of annealing at 57°C, 45secs at 72°C, and a final step for 5mins at 72°C. Each PCR was performed with a negative control of ddH20 and a positive control from a mite population in our laboratory that was previously reported to be infected with an endosymbiont. For *Wolbachia*, DNA from five adult female *Bryobia* mites was extracted by crushing individuals in 100µl of a 5% Chelex solution with 10µl of 20mg/ml proteinase K; samples were placed in a thermocycler at 37°C for 30mins followed by 7mins at 95°C. For *Cardinium* and *Spiroplasma*, the DNA of four adult *Brevipalpus* mites and of four adult *T. urticae* females, respectively, was extracted using the same Chelex-based protocol. Amplicons were run on 1% agarose gels for ~45mins at 120V.

Second, we sequenced the bacterial 16S rDNA subunit from a 1:10 DNA aliquot from each of our 26 iso-female lines, plus three control populations. We used the same phenol-based extraction protocol as described above to obtain purified DNA from three other *T. urticae* laboratory populations previously determined to be infected by *Wolbachia*, *Spiroplasma* and *Cardinium*, as positive controls. 16S rDNA was amplified and sequenced by LGC Genomics, (Germany), using an Illumina MiSeq platform. Briefly, DNA samples were checked for quality, and PCR was targeted for the V3-V4 region of the 16s subunit, according to the MiSeq Illumina guidelines. PCR products were cleaned of remaining primers and primer dimers, after which Nextera adapter sequences were attached to the amplicons of each sample. A second round of PCR clean-up was used to produce the normalised libraries used for sequencing. Around 5 million read pairs were generated (~100 000 reads per sample, 2X300bp), which were then

processed according to the Qiime2 pipeline [3] using custom Python scripts (miniconda3 environment). Raw fastaq files were demultiplexed by barcode identity. The quality of the sequence data was further controlled using the dada2 pipeline for Illumina reads. The taxonomic analysis was performed using a Naïve Bayes classifier based on the Greengenes 13_8 99% OTUs from 515F/806R region of sequences. Bar graphs representing the relative bacterial taxa frequencies per sample were obtained with the 'Qiime2 view' interface.

Supplementary Note 4: Mapping and variant calling

Illumina genomic library construction and DNA sequencing were performed at the High-throughput Genomics Core at the Huntsman Cancer Institute of the University of Utah (Salt Lake City, USA) to produce paired-end reads of 125 bp as previously described [4]. The resulting reads were aligned to the *T. urticae* reference genome [5] using the default settings of BWA 0.7.15-r1140 [6] and sorted by coordinate using SAMtools 1.3.1 [7]. In line with the recommendations described in the GATK Best Practices workflow [8] duplicate reads were marked with Picard tools 2.6.0-SNAPSHOT [https://broadinstitute.github.io/picard] prior to indel realignment with GATK 3.6-0-g89b7209 [9]. SNPs were then called jointly across these and select other *T. urticae* strains for which Illumina sequences were previously deposited in public databases [see BioProject PRJNA530192, and Bryon *et al.* [10], Snoeck *et al.* [11], and Wybouw *et al.* [4,12]; collectively, strains Albino-JP, Brazil, Catnip6, FG, GH, Heber, Hib, Foothills, KH, Kigen, Lemon5, London Inbred, MAR-AB, MR-VL, MR-VP, NightS, PA2, ParkCity, Parrott, PyrR, RB, RS, ShCo, Spain, SR-VP, TuSB9, UK, Wasatch, WG-Del, WG-S] using GATK UnifiedGenotyper [9]. Samples released in PRJNA530192 but that potentially do not belong to *T. urticae* were only included as well in the joint variant call if we were able to identify them to species using ITS2 sequences, please see "Species identification based on ITS sequences" below, as assessed with the output of the GATK UnifiedGenotyper tool.

Supplementary Note 5: Species identification based on ITS2 sequences

Some descriptions for *Tetranychus* strains submitted as part of BioProject PRJNA530192 noted a high level of divergence from *T. urticae*, and the strains were flagged as potential cryptic species or sister species. To assign species identifications to these strains where possible, we examined their ITS2 sequences in the Illumina read alignments to the *T. urticae* genome (see section "mapping and variant calling"). To determine the location of the ITS2 repeats in the *T. urticae* assembly, we aligned the *Tetranychus* ITS2 primers used for phylogenetic analysis in Ben-David *et al.* [13] to the *T. urticae* 640-scaffold assembly using ORCAE [14], which placed the repeats in several locations along scaffold 42. We then used the Integrative Genomics Viewer (IGV) v. 2.3 [15] to determine the location of variants in the repeats. As reads did not align uniquely in the repeats, and read coverage was variable, we focused on the sequence between positions 100,363 and 100,883 on scaffold 42 where the read coverage was relatively even. Using this method, we were able to identify strains GD, Jriv, Mt0 and Sh, as belonging to *T. turkestani*, as they matched the consensus *T. turkestani* ITS2 sequence based on previously reported sequences of ITS2 fragments [16]. Strains that did

not match any of the previously described ITS2 patterns were not included in the joint variant call described in the previous section.

Supplementary Note 6: Quality control on predicted variants

Variants predicted by GATK were subjected to quality control (QC), and those that passed were considered for further analyses; the QC metrics we used on the output variant call format (VCF; v. 4.2) were adapted from recommendations in GATK's post #2806 (https://gatkforums.broadinstitute.org/gatk/discussion/2806/howto-apply-hard-filters-to-a-call-set, accessed 9 July 2018) and required that each SNP had (1) a quality score normalized by allele depth (QD field in the VCF file) of at least 2, (2) mean root square mapping quality (MQ) of at least 50, (3) strand odds ratio (SOR) below 3, (4) mapping quality rank sum (MQRankSum) higher than or equal to -8, (5) rank sum for relative positioning of reference versus alternative alleles in reads (ReadPosRankSumTest) of at least -8, and also (6) fall within 25% and 150% of the sample's genome-wide mean SNP read coverage (AD). These QC metrics were used for all analyses unless specified otherwise.

Supplementary Note 7: Levels of heterozygosity

To avoid counting SNPs in copy-variable regions as heterozygotes, QC settings were adjusted from those outlined in *Quality control on predicted variants.* Specifically, only SNPs with coverage depth falling within 0.75x and 1.25x of the genome-wide mean were considered, and mapping quality rank sum score (MQRankSum), as well as rank sum for relative positioning of reference versus alternative alleles in reads (ReadPosRankSumTest) had to be within -8 and 8. Percentage of heterozygous SNPs across the genome were determined in 100kb sliding windows with a 10kb offset. Each window had to contain at least 20 SNPs to be included.

Supplementary Note 8: Genome-wide analysis of SNP similarity between lines

SNPs that passed quality control (as per *A6*) were used to calculate genome-wide percent SNP similarity between the lines. If a SNP was heterozygous, we chose the allele with the higher Illumina read support (using the AD field in the VCF file). Percent similarity was then computed using custom Python scripts with the help of the Python package pandas v. 1.0.3 [17].

Supplementary References

1 Gotoh, T., Noda, H. & Ito, S. Cardinium symbionts cause cytoplasmic incompatibility in spider mites. *Heredity* **98**, 13-20 (2007).

2 Fukatsu, T., Tsuchida, T., Nikoh, N. & Koga, R. *Spiroplasma* symbiont of the pea aphid, *Acyrthosiphon pisum* (Insecta: Homoptera). *Applied and Environmental Microbiology* **67**, 1284-1291, doi:10.1128/AEM.67.3.1284-1291.2001 (2001).

3 Bolyen, E. *et al.* Reproducible, interactive, scalable and extensible microbiome data science using QIIME 2. *Nature Biotechnology* **37**, 852-857, doi:10.1038/s41587-019-0209-9 (2019).

4 Wybouw, N. *et al.* Long-term population studies uncover the genome structure and genetic basis of xenobiotic and host plant adaptation in the herbivore *Tetranychus urticae*. *Genetics* **211**, 1409-1427, doi:10.1534/genetics.118.301803 (2019).

5 Grbic, M. *et al.* The genome of *Tetranychus urticae* reveals herbivorous pest adaptations. *Nature* **479**, 487-492, doi:http://www.nature.com/nature/journal/v479/n7374/abs/nature10640.html#supplementary-information (2011).

6 Li, H. Aligning sequence reads, clone sequences and assembly contigs with BWA-MEM. *arXiv preprint arXiv:1303.3997* (2013).

7 Li, H. *et al.* The Sequence Alignment/Map format and SAMtools. *Bioinformatics* **25**, 2078-2079, doi:10.1093/bioinformatics/btp352 (2009).

8 Van der Auwera, G. A. *et al.* From fastq data to high-confidence variant calls: the genome analysis toolkit best practices pipeline. *Current Protocols in Bioinformatics* **43**, 11.10.11-11.10.33, doi:10.1002/0471250953.bi1110s43 (2013).

9 DePristo, M. A. *et al.* A framework for variation discovery and genotyping using next-generation DNA sequencing data. *Nat Genet* **43**, 491-498, doi:10.1038/ng.806 (2011).

10 Bryon, A. *et al.* Disruption of a horizontally transferred phytoene desaturase abolishes carotenoid accumulation and diapause in *Tetranychus urticae*. *Proceedings of the National Academy of Sciences* **114**, E5871-E5880, doi:10.1073/pnas.1706865114 (2017).

11 Snoeck, S. *et al.* High-resolution QTL mapping in *Tetranychus urticae* reveals acaricide-specific responses and common target-site resistance after selection by different METI-I acaricides. *Insect Biochemistry and Molecular Biology* **110**, 19-33 (2019).

12 Wybouw, N. *et al.* Convergent evolution of cytochrome P450s underlies independent origins of keto-carotenoid pigmentation in animals. *Proceedings of the Royal Society B* **286**, 20191039, doi:10.1098/rspb.2019.1039 (2019).

13 Ben-David, T., Melamed, S., Gerson, U. & Morin, S. ITS2 sequences as barcodes for identifying and analyzing spider mites (Acari: Tetranychidae). *Experimental and Applied Acarology* **41**, 169-181, doi:10.1007/s10493-007-9058-1 (2007).

14 Sterck, L., Billiau, K., Abeel, T., Rouzé, P. & Van de Peer, Y. ORCAE: online resource for community annotation of eukaryotes. *Nature Methods* **9**, 1041-1041, doi:10.1038/nmeth.2242 (2012).

15 Robinson, J. T. *et al.* Integrative genomics viewer. *Nature Biotechnology* **29**, 24-26, doi:10.1038/nbt.1754 (2011).

16 Navajas, M. & Boursot, P. Nuclear ribosomal DNA monophyly versus mitochondrial DNA polyphyly in two closely related mite species: the influence of life history and molecular drive. *Proceedings of the Royal Society B* **270 Suppl 1**, S124-127, doi:10.1098/rsbl.2003.0034 (2003).

17 McKinney, W. in *Proceedings of the 9th Python in Science Conference.* 51-56 (Austin, TX).
