# Supplementary material for: Adaptive divergence and post-zygotic barriers to gene flow between sympatric populations of a herbivorous mite
Source: Commun Biol. 2021 Jul 9;4:853. doi: 10.1038/s42003-021-02380-y (PMC8270941; doi:10.1038/s42003-021-02380-y)
Supplement: Supplementary file 3 — Life sciences reporting summary [file 42003_2021_2380_MOESM3_ESM.pdf]

nature research

Corresponding author(s): Ernesto Villacis-Perez
Johannes Breeuwer
Thomas van Leeuwen

Last updated by author(s): May 20, 2021

Reporting Summary

Nature Research wishes to improve the reproducibility of the work that we publish. This form provides structure for consistency and transparency in reporting. For further information on Nature Research policies, see our Editorial Policies and the Editorial Policy Checklist.

Statistics

For all statistical analyses, confirm that the following items are present in the figure legend, table legend, main text, or Methods section.

n/a	Confirmed	
☐	☒	The exact sample size (n) for each experimental group/condition, given as a discrete number and unit of measurement
☐	☒	A statement on whether measurements were taken from distinct samples or whether the same sample was measured repeatedly
☐	☒	The statistical test(s) used AND whether they are one- or two-sided<br>*Only common tests should be described solely by name; describe more complex techniques in the Methods section.*
☒	☐	A description of all covariates tested
☐	☒	A description of any assumptions or corrections, such as tests of normality and adjustment for multiple comparisons
☐	☒	A full description of the statistical parameters including central tendency (e.g. means) or other basic estimates (e.g. regression coefficient) AND variation (e.g. standard deviation) or associated estimates of uncertainty (e.g. confidence intervals)
☐	☒	For null hypothesis testing, the test statistic (e.g. F, t, r) with confidence intervals, effect sizes, degrees of freedom and P value noted<br>*Give P values as exact values whenever suitable.*
☒	☐	For Bayesian analysis, information on the choice of priors and Markov chain Monte Carlo settings
☒	☐	For hierarchical and complex designs, identification of the appropriate level for tests and full reporting of outcomes
☒	☐	Estimates of effect sizes (e.g. Cohen's d, Pearson's r), indicating how they were calculated

Our web collection on statistics for biologists contains articles on many of the points above.

Software and code

Policy information about availability of computer code

Data collection

No software was used to collect the data in this study

Data analysis

The following software and packages were used throughout this study. More details about their functionality and implementation can be found in the Material and Methods and Appendix section.
CodonCode Aligner (v. 6.0.2)
MEGA for Mac (v. 7)
BWA (v. 0.7.12-r1039)
MAFFT (v. 7.7217)
PartitionFinder2
RAxML (v. 8.2.8)
PhyML (v. 3.1)
Seaview (v. 4.6.1)
R (v. 3.3.2)
R-packages: SNPRelate (v. 1.8.0), gdsfmt (v. 1.10.1), PopGenome (v. 2.6.1), lme4 (v1.1-7)
BWA (v. 0.7.15-r1140)
SAMtools (v 1.3.1)
Picard tools (v 2.6.0)
GATK (v. 3.6-0-g89b7209)
ORCAE
Integrative Genomics Viewer (v. 2.3)
Python 3 (v3.4) packages:
pandas (v. 1.0.3)

numpy (1.16.2)
Matplotlib (v. 3.1.1)

Heterozygosity levels were calculated using custom Python 3 scripts available at github.com/akurlovs/hetero

For manuscripts utilizing custom algorithms or software that are central to the research but not yet described in published literature, software must be made available to editors and reviewers. We strongly encourage code deposition in a community repository (e.g. GitHub). See the Nature Research guidelines for submitting code & software for further information.

Data

Policy information about availability of data

All manuscripts must include a data availability statement. This statement should provide the following information, where applicable:

- Accession codes, unique identifiers, or web links for publicly available datasets
- A list of figures that have associated raw data
- A description of any restrictions on data availability

Sequence data that support the findings of this study have been deposited in the following databases:
CO1 sequences in GenBank with the accession codes MT814055-MT814210
Whole genome sequences of field-derived mite lines in SRA with accessions SAMN13693727-52
16S bacterial sequences of field-derived mite lines in SRA with accessions SRR12491964-89 and of laboratory lines with accessions SRR12492212-14
Whole genome sequencing of Tetranychus urticae green and red morphs,and T. turkestani, T. kanzawai sequences in SRA under bioproject PRJNA530192.
Datasets used to generate Figures 1, 4, 5, Supplementary Figures 2, 8 and 9, and Supple-mentary Tables 2 and 3 are available in figshare with the identifier https://doi.org/10.21942/uva.c.5447778.v1.
Additional datasets that support the findings of this study are available from the corresponding author upon reasonable request.

Field-specific reporting

Please select the one below that is the best fit for your research. If you are not sure, read the appropriate sections before making your selection.

☐ Life sciences ☐ Behavioural & social sciences ☒ Ecological, evolutionary & environmental sciences

For a reference copy of the document with all sections, see nature.com/documents/nr-reporting-summary-flat.pdf

Ecological, evolutionary & environmental sciences study design

All studies must disclose on these points even when the disclosure is negative.

Study description	We investigated intra-specific differences within and between populations of the herbivorous spider mite Tetranychus urticae. We sampled and genotyped individual adult mites found in two separate locations along the Dutch dune ecosystem by amplifying and sequencing a mitochondrial marker (CO1), across three field seasons, from 2015 to 2017, from a total of 1023 individuals. From these individuals, 157 unique haplotypes were retrieved and used for phylogenetic analyses. Twenty-six iso-female lines were created in the laboratory. Whole genome sequencing was used to investigate the extent of genome-wide differentiation between them field-derived lines, plus 30 additional genomes of this species (28 of the green morph of this species, 2 of the red morph) and 5 genomes of closely related species T. turkestani (4 genomes) and T. kanzawai (1 genome). The resulting dataset was used to perform the genomic analyses. Amplification of 16S rDNA bacterial sequences from the 26 field-derived lines plus three laboratory lines were performed prior to crossing assays. Three independent crossing assays were performed, each with a pair of field-derived lines representing three common mite genotypes found in the field, with approximately 50 crosses per treatment in total. Differences between crossing treatments and intraline controls were compared using linear mixed models, with 'crossing treatment' as fixed effect, and isofemale line and experimental block as random effects. Quantification of fitness traits of field-derived lines on different plant species were compared between lines. Reproductive output per unit of time between genotypes was compared for each of 5 different host plants using a linear mixed model with genotype as fixed effect, and the iso-female line as random factor. Juvenile survival was quantified as a proportion of surviving individuals per replicate on honeysuckle for 6 lines in three independent experiments, each with a different pair of mite lines and compared using a linear mixed model with line as the fixed factor and the side of the leaf and each replicate per genotype as random factors.
Research sample	Field populations and derived laboratory lines of the two-spotted spider mite Tetranychus urticae that occurred naturally in the dune ecosystem of The Netherlands. Juvenile and adult individuals from these populations were collected from the field and used in different experiments. Additional laboratory populations of T. urticae green and red morphs, T. kanzawai and T. turkestani were originally collected by RMC and collaborators
Sampling strategy	Field sampling was performed extensively and there were no predeterminations of sample sizes needed. During the 2015 season, 160 individuals were successfully genotyped. For the 2016 season, 210 individuals were successfully genotyped. For the 2017 season, a new DNA extraction and amplification protocol was used and 627 individuals were successfully genotyped. Every season the same patterns were observed, and therefore the sample size of 2015 was deemed sufficient. The number of samples used for whole genome sequencing were based on financial and technological limitations.
Data collection	Field sampling, genotyping and laboratory assays were perfomed by EV-P. In the field, the coordinates of each population, the host plant on which the individuals were found and the date of collection were annotated. DNA extraction for the field-derived lines was performed by EV-P. Laboratory experiments were performed at the University of Amsterdam by EV-P and supervised by JB and TVL. Genome sequencing of laboratory populations was performed by RMC and collaborators.

Timing and spatial scale	Field sampling was performed in the summers of 2015, 2016 and 2017. Sampling occurred between July and August every year. During this period, spider mite populations are large enough to be established on their hosts, but not advanced enough that the plant is destroyed. This study was designed to extensively sample a small spatial scale, comprised by two separate sampling sites in the Dutch dune ecosystem. Within each of these sites, transects were traced along forested areas and sampling locations were established at least 50 meters apart from each other. Within each of these locations, all the plants found to be infested by mites in a 10m2 area were sampled and brought back to the laboratory for processing within a maximum of 48 hours from collection.
Data exclusions	No data were excluded from the study
Reproducibility	Field sampling was reproduced every season across three consecutive years. Laboratory assays (crossing assays and fitness quantification) were performed in batches of three experiments using different iso female lines to ensure reproducibility.
Randomization	Field sampling was done extensively and multiple individuals were sampled. Adult mites used for mitochondrial genotyping were selected randomly from each host plant from each sampling location. Field-derived lines were started from selected individuals that belonged to the genotypes previously described by the field sampling.
Blinding	Blinding was not relevant for this study, since we are primarily describing a natural system using an experimental design that is not biased by the observations of each researcher

Did the study involve field work? ☒ Yes ☐ No

Field work, collection and transport

Field conditions	Field sampling was performed across three consecutive years in the Dutch dunes. Each year, sampling was performed during July/ August, when rainfall did not occur, and the temperature was on average 25C.
Location	All sampling was performed in the Dutch dune ecosystem located near to Castricum and Meijendel in The Netherlands, which are at sea level.
Access & import/export	All samples were obtained from public natural areas under permission of the relevant authorities of the natural areas in the Netherlands, namely PWN for Castricum and Dunea in Meijendel.
Disturbance	Sampling of commonly occurring plants infested by mites was not destructive; only a reduced number of leaves were collected.

Reporting for specific materials, systems and methods

We require information from authors about some types of materials, experimental systems and methods used in many studies. Here, indicate whether each material, system or method listed is relevant to your study. If you are not sure if a list item applies to your research, read the appropriate section before selecting a response.

Materials & experimental systems

n/a	Involved in the study	
☒	☐	Antibodies
☒	☐	Eukaryotic cell lines
☒	☐	Palaeontology and archaeology
☐	☒	Animals and other organisms
☒	☐	Human research participants
☒	☐	Clinical data
☒	☐	Dual use research of concern

Methods

n/a	Involved in the study	
☒	☐	ChIP-seq
☒	☐	Flow cytometry
☒	☐	MRI-based neuroimaging

Animals and other organisms

Policy information about studies involving animals; ARRIVE guidelines recommended for reporting animal research

Laboratory animals	We developed field-derived laboratory lines of the two-spotted spider mite Tetranychus urticae
Wild animals	Field sampling consisted on identifying plants infested with mites and collecting several infested leaves wrapped in kitchen paper and placed inside individual plastic bags. After a max of 48h, samples were analyzed under the microscope and 10 individuals per plant per location were collected for DNA extraction and genotyping. The remaining leaves and individuals were discarded in a biological material container.
Field-collected samples	In 2015, populations representing three common mite genotypes were sampled and used to establish iso-female lines in the laboratory. Twenty six iso-female lines were created, each from a single virgin female crossed to her son and kept since then on detached common bean leaves (Phaseolus vulgaris) surrounded by wet cotton wool to prevent cross contamination. All lines were kept since then in a room with controlled climate conditions (25C, light:dark period of 16h:8h and 60% relative humidity).
Ethics oversight	We study commonly occurring invertebrates for which no ethical guides exist

Note that full information on the approval of the study protocol must also be provided in the manuscript.
